# Supplementary material for: A high-resolution mRNA expression time course of embryonic development in zebrafish
Source: eLife. 2017 Nov 16;6:e30860. doi: 10.7554/eLife.30860 (PMC5690287; doi:10.7554/eLife.30860)
Supplement: Supplementary file 6. [file elife-30860-supp6.zip › biolayout-clusters-files/Cluster227-genes.html]

Cluster227


# Cluster227: Genes

| Ensembl ID | Gene Name | Chr | Start | End | Biotype |
| --- | --- | --- | --- | --- | --- |
| ENSDARG00000069356 | adgrl2a | 11 | 7518760 | 7727096 | protein\_coding |
| ENSDARG00000019526 | agfg1b | 2 | 16791731 | 16810701 | protein\_coding |
| ENSDARG00000075819 | arhgef2 | 16 | 6923930 | 6992500 | protein\_coding |
| ENSDARG00000035178 | gna14 | 5 | 54873358 | 54890801 | protein\_coding |
| ENSDARG00000033466 | tagln2 | 5 | 36487430 | 36495307 | protein\_coding |
| ENSDARG00000102464 | wnt5b | 4 | 8531664 | 8662201 | protein\_coding |
